# Predicting Critical Loads in Fused Deposition Modeling Graphene-Reinforced PLA Plates Containing Notches Using the Point Method

**DOI:** 10.3390/polym15183797

**Published:** 2023-09-18

**Authors:** Sergio Cicero, Marcos Sánchez, Sergio Arrieta

**Affiliations:** LADICIM, Departamento de Ciencia e Ingeniería del Terreno y de los Materiales, Universidad de Cantabria, Avenida de los Castros, 44, 39005 Santander, Spain; marcos.sanchez@unican.es (M.S.); sergio.arrieta@unican.es (S.A.)

**Keywords:** fracture, fused deposition modeling, graphene, PLA, notch, point method

## Abstract

The use of 3D-printed composites in structural applications beyond current prototyping applications requires the definition of safe and robust methodologies for the determination of critical loads. Taking into account that notches (corners, holes, grooves, etc.) are unavoidable in structural components, the presence of these types of stress risers affects the corresponding load-carrying capacity. This work applies the point method (PM) to the estimation of the critical (fracture) loads of graphene-reinforced polylactic acid (PLA-Gr) plates obtained via fused deposition modeling (FDM) with a fixed raster orientation at 45/−45. Additionally, the plates contain three different notch types (U-notches, V-notches, and circular holes) and comprise various thicknesses (from 5 mm up to 20 mm) and ratios of notch length to plate width (a/W= 0.25 and a/W = 0.50). The comparison between the obtained experimental critical loads and the corresponding estimations derived from the application of the PM reveals that this approach generates reasonable accuracy in this particular material that is comparable to the accuracy obtained in other structural materials obtained via traditional manufacturing processes.

## 1. Introduction

Fused deposition modeling (FDM), framed within the category of additive manufacturing (AM) technologies, allows intricate geometries to be fabricated. Moreover, FDM is applicable to a wide variety of materials, covering polymers, metals, ceramics, and composites. FDM basically consists of extruding a melted filament through a heated nozzle; this filament is then deposited on a build platform layer by layer until the final component is generated [1]. Herein, although there may be some nuances, FDM and fused filament fabrication (FFF) will be considered the same technology. To date, FDM has essentially been used for prototyping but not to generate components with structural purposes, since the mechanical properties obtained via FDM are commonly lower than those attained through the use of more traditional fabrication technologies, such as injection molding or extrusion. However, aiming to improve the mechanical performance of FDM materials, there have been significant research efforts to generate improved knowledge of this technique and the mechanical behaviors of the resulting printed materials (e.g., [1,2,3,4,5]).

Different strategies have been proposed with the aim of improving the material performance of polymer–matrix composites, among which the addition of nano-reinforcements (e.g., carbon nanotubes [6], silica nanoparticles [7], nano-clay [8], graphene [9], or graphene oxide (GO) [10,11]) is of great interest. A general observation is that the different works normally present an enhancement in the material properties being analyzed in each case, although negative results have also been reported (e.g., [12]). In this sense, graphene and its derivatives (e.g., graphene oxide) have been broadly applied as reinforcing materials over the last decade, given that they present excellent mechanical properties that may greatly improve the mechanical performance of the matrix. The investigation of the mechanical behavior of composites reinforced with graphene, including FDM materials, has focused mainly on their tensile properties (e.g., [13,14,15,16,17]) and, to a lesser extent, on fracture behavior (e.g., [18]) in the presence of defects.

On the other hand, structural components usually contain stress risers (herein referred to as notches), and materials obtained through FDM are not an exception, as they may contain defects produced during fabrication (e.g., pores), geometrical details included in their design (e.g., holes, grooves, or corners), or defects caused via operational damage, among others. These defects are not crack-like defects, so it may be overly conservative to proceed on the hypothesis that they behave like cracks and to apply conventional fracture mechanics criteria. Thus, the evaluation of their effect on structural integrity demands specific approaches beyond conventional fracture mechanics analyses. Among the different approaches that may be applied to analyze notches, the point method (PM) [19,20] provides an excellent balance between the simplicity of the analysis and the accuracy of its predictions. The PM has been widely applied and validated in conventional structural materials containing notches (e.g., [20,21,22,23,24]), although its application to FDM notched materials has been scarce (e.g., [4,18,24,25,26]), especially when dealing with graphene-reinforced FDM notched materials [18]. Similarly, most of the applications have been performed on notched fracture mechanics specimens (e.g., CT specimens or SENB specimens) (e.g., [18,21,26]), with a much smaller number of works dealing with geometries that are generally closer to real structural parts (e.g., [4,23,24,27]), as is the case of notched plates. The proper PM has shown its capacity to distinguish between defect sizes that affect the load-bearing capacity of the corresponding structural part and small defects that do not directly affect the structural performance of the part [19].

Taking this into consideration, the present work provides an approach to analyzing the structural integrity of FDM-generated graphene-reinforced PLA (PLA-Gr) structural plates containing macroscopic notches such as those derived from geometrical details or operational damage. The analysis of microscopical defects, such as those that may be generated during the fabrication process, is beyond the scope of the present research. Thus, Section 2 provides a description of the basic characterization of the PLA-Gr material being analyzed, the experimental program performed on the PLA-Gr notched plates, and the point method itself, which was applied to derive the corresponding critical loads. Section 3 gathers the results, comparing the fracture load estimations with the corresponding experimental results, and provides a discussion, and Section 4 summarizes the main conclusions.

## 2. Materials and Methods

### 2.1. Material

The material analyzed in this work was an FDM-generated graphene-reinforced (1 wt%) PLA (PLA-Gr), which was supplied by FiloAlfa3D (Turin, Italy) as filaments for use in the printing process. In a previous work [18], the authors provided a complete basic characterization (characterizing both the tensile and fracture properties) of the PLA-Gr material obtained via three different raster orientations (0/90, 30/−60, and 45/−45); they also provided the same characterization for the parent FDM-generated PLA material.

The different tensile and fracture specimens were all manufactured using a Prusa i3 printer (Prusa, Prague, Czech Republic) with the following printing parameters: a nozzle diameter of 0.4 mm; a nozzle temperature of 200 °C; a bed temperature of 75 °C; a printing rate of 30 mm/s; an infill level of 100%; and a layer height of 0.3 mm. Concerning the obtained results, it can be noted that the raster orientation of 45/−45 was significantly more sensitive to the addition of graphene than the 0/90 and 30/−60 raster orientations, developing noticeably higher tensile strength and fracture toughness values when adding the nano-reinforcement compared to those developed via the simple FDM-generated PLA material. The main mechanical properties for the 45/−45 materials (with and without graphene) are summarized in Table 1 [18], where E is the elastic modulus, σ_y_ is the yield stress, σ_u_ is the ultimate tensile strength, ε_u_ is the strain under maximum load, and K_mat_ is the material fracture toughness. The tensile properties were obtained using dog bone specimens (three per material, PLA and PLA-Gr), following the ASTM638 standard [28], whereas the fracture toughness was determined using single-edge notched bending specimens (four per material), following the ASTM D6068 standard [29]. As the notched plates described below were tested with the same loading orientation regarding the printing direction as that used in the tensile and fracture characterization specimens, the moderate anisotropy associated with this type of material will not be considered in this work (e.g., see [4] for experimental evidence in this regard). This anisotropy might need to be considered in the case of subjecting the 45/−45 material to other loading directions. Nonetheless, the reader is referred to the original article for a more detailed description of the results and the experimental processes.

### 2.2. Experimental Program

Once the basic material mechanical properties were defined, the same PLA-Gr material was used to generate 51 plates that combined three types of macroscopic notches (U-notches, V-notches, and circular holes), 2 different nominal notch radii (ρ = 0.9 mm and 1.3 mm), two nominal specimen widths (W = 60 mm and 120 mm), various specimen thicknesses (B = 5 mm, 10 mm, and 20 mm), and two notch length to specimen width ratios (a/W = 0.25 and 0.50). As a result, there were 17 distinct geometries and three specimens per geometry. The summary of the geometries is as follows.

- 27 U-notched specimens with (nominal) notch radii of 0.9 mm or 1.3 mm, a/W = 0.25 or 0.50 (a being the notch length and W being the specimen width), and 3 different thicknesses (5 mm, 10 mm, and 20 mm). The notch radius of 0.9 mm covered all 3 thicknesses, while the 1.3 mm notch radius was only applied to 5-mm- and 10-mm-thick plates.
- 12 V-notched plates: V-notch with opening angle of 60°, nominal notch radius of 0.9 mm or 1.3 mm, a/W = 0.25 or 0.50, and 2 different thicknesses (5 mm and 10 mm).
- 12 plates with circular holes: nominal notch radius, ρ (or circle radius, a), fixed at 15 mm, a/W = 0.25 or 0.50, and 2 different thicknesses (5 mm and 10 mm).

**Figure 1:**
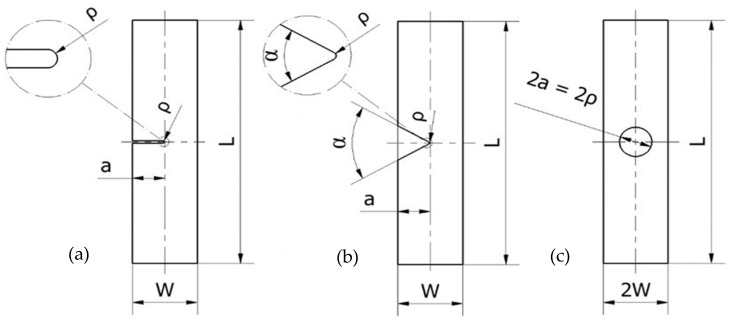
Geometries of the tested specimens. (**a**) U-notched specimens; (**b**) V-notched specimens; (**c**) specimens with central hole. The thickness varied from 5 mm up to 20 mm.

Figure 1 shows a schematic of the tested plates, while the whole experimental program is presented in Table 2. It may be noted that the actual values of the different geometrical parameters were physically measured, as in some cases they were moderately different from the nominal ones.

All the plates were printed with the same printer and the same printing parameters used in the basic (tensile and fracture) characterization (described in Section 2.1 [18]).

Finally, it is important to note that the notches were machined and not printed. The reason for this practice is the better finishing of machined defects when compared to FDM-printed ones [27]. The latter, additionally, generate an orientation of the filaments around the fracture process zone that may significantly affect the fracture process.

**Figure 2:**
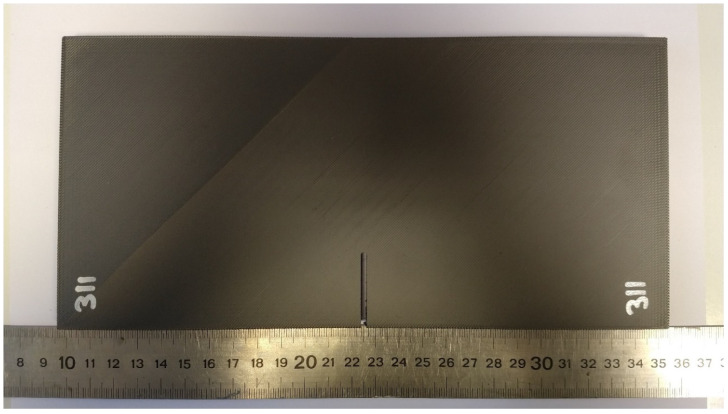
Specimen G311, with nominal values of a/W = 0.25, W = 60 mm, ρ = 1.3 mm, and B = 10 mm (nominal values).

Figure 2 shows one of the specimens that was part of the experimental program. Once fabricated, all the plates were subjected to tensile loading in a servo-hydraulic testing machine (INSTRON, Norwood, MA, USA). The displacement rate was 1 mm/min in all cases (again, the same displacement rate used in the basic characterization). The load–displacement curve was recorded for each individual test, also determining the corresponding critical (i.e., maximum) load.

### 2.3. Analytical Approach

The PM was applied with the aim of estimating the critical loads of the different plates. The PM is one of the methodologies included within the Theory of Critical Distances (TCD) and, thus, it is characterized by the use of a material length parameter (the critical distance, L). The origins of the TCD date back to the middle of the twentieth century [30,31], although it has been widely established and validated in the last two decades (e.g., [4,21,22,23,24,25,26]). The main parameter of the TCD, the critical distance (L), follows Equation (1):

L = (1/π)·(K_mat_/σ_0_)^2^ (1)

with K_mat_ being the fracture toughness and σ_0_ being the material’s inherent strength. The value of the inherent strength (σ_0_) is the material ultimate tensile strength (σ_u_) in those materials with linear–elastic behavior at both the micro and the macro scales, whereas in materials with non-fully linear behavior, σ_0_ requires calibration [19]. The calibration for the PLA-Gr material and raster orientation analyzed in this work was performed in [18] through the best fit of the PM predictions of the experimental apparent fracture toughness values obtained in SENB specimens containing notches with different notch radii (see [18] for details). The values of L and σ_0_ were 0.48 mm and 185.4 MPa, respectively (note that σ_0_ was much larger than σ_u_).

**Figure 3:**
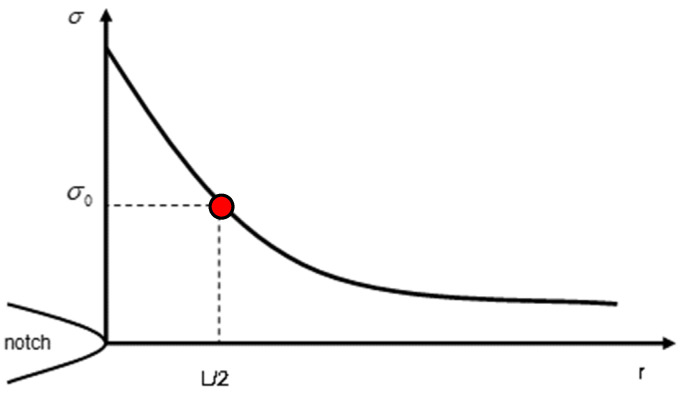
Definition of the point method approach.

The PM is the simplest version of the TCD, and it is based on the stress field at the defect tip being analyzed. It assumes that a fracture takes place when the stress reaches the inherent stress, at a distance of L/2 from the defect tip (see Figure 3). The resulting failure criterion is

σ(L/2) = σ_0_ (2)

Thus, once the PM parameters (L and σ_0_) are known, the application of this approach requires the definition of the stress field ahead of the notch tip. With this aim, all the specimens, with their particular dimensions, were modeled in the finite element (FE) software (Ansys 2023 R1) via 20-node hexahedral elements and linear–elastic behavior (see Table 1), determining, for an arbitrary external tensile load (1N in this case), the stress field in the mid-plane of the fracture section and the corresponding stress value at a distance of L/2. Then, the critical load was determined by applying proportionality conditions, as the one generating a stress value equal to σ_0_ at the same distance (L/2), as established by the PM at fracture conditions. Here, it is important to note that, given that the stress field may be highly sensitive to the specimen geometry (especially the notch radius), and taking into account that the actual values of the geometrical parameters may differ moderately from the nominal values, the simulation was performed for every tested specimen. The results, shown below, reveal that this practice was only necessary for V-notched specimens, given that the variation in the estimated critical load for U-notched specimens and specimens with a central hole was basically negligible.

In order to optimize the computational cost, the analysis was performed using 2D models and plane stress conditions, resulting in an average number of elements on each plate of approximately 30,000. These assumptions were previously validated by developing 3D models on a limited number of specimens, yielding very similar results. Additionally, and because of the symmetry conditions, the models (notched plates) were sectioned along their respective planes of symmetry, establishing the corresponding fixed (null) displacements along them (see Section 3). Given the strong dependency of the stress at the notch tip on the mesh size, the mesh was refined at the notch tip, resulting in element sizes in the order of 0.01 mm. It is noteworthy that the element sizes were maintained well below the calculated L (0.48 mm).

## 3. Results and Discussion

**Figure 4:**
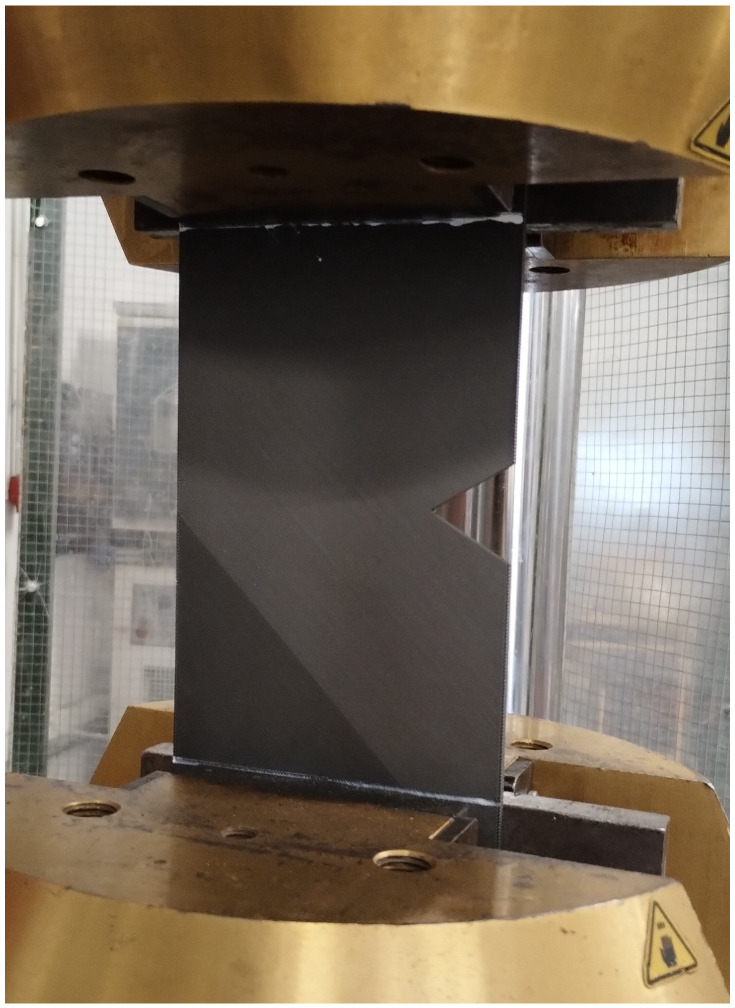
Example of experimental setup. V-notched specimen G407.

**Figure 5:**
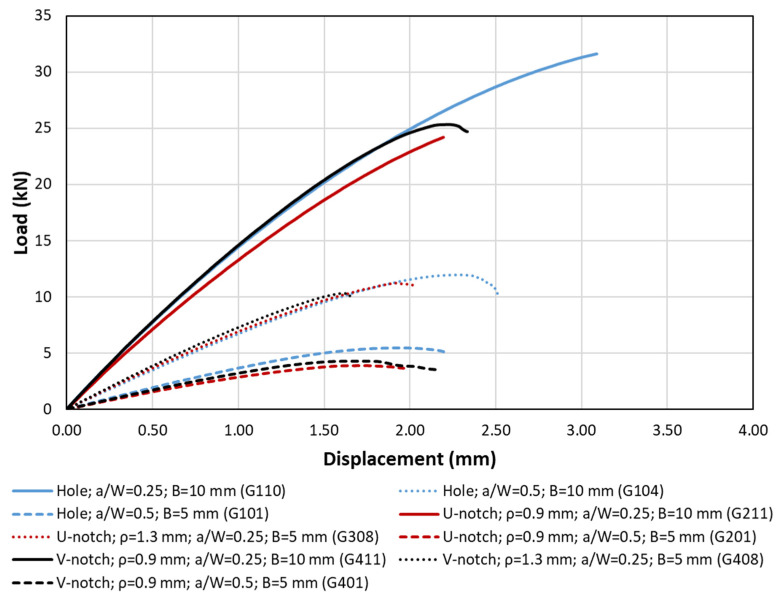
Examples of load–displacement curves for different specimen geometries.

Figure 4 and Figure 5 show, respectively, examples of the experimental setup and the obtained load–displacement curves, while Table 2 gathers the experimental critical loads as well as the corresponding estimations provided by the PM (P_PM_). The top three curves shown in Figure 5 (solid lines) correspond to 10-mm-thick plates and a/W equal to 0.25, with the only difference being the geometry of the corresponding defect (U-notch, V-notch, and hole). Both the U-notch and the V-notch have a notch radius of 0.9 mm. It can be observed that the initial slope is coincident in the three curves, with the curve corresponding to the U-notch deviating from the others in the early stage of the loading process (at 5 kN, approximately) and leading to a lower critical load after a more linear–elastic process than those observed in the other cases. The curves for the V-notched plate and the plate with a central hole are coincident up to a load level of approximately 24 kN. At this point, the V-notched plate develops non-linear behavior with a progressively lower slope until the final fracture. In contrast, the plate with a central hole suffers from a very smooth, progressive loss of slope, with less evident non-linear behavior, and achieves the highest critical load of the three geometries.

Concerning the dashed lines (i.e., lower curves), the observations are analogous. They all correspond to 5-mm-thick specimens with a/W = 0.5, the difference being restricted to the type of defect. Again, the initial slope is coincident in the three curves, although, in this case, they progressively separate from each other throughout the whole loading process. As in the previous case, the U-notched plate led to a lower critical load, with the plate containing the hole developing the highest critical load.

Finally, the dotted lines represent 5-mm-thick plates with a/W = 0.25. The behavior is analogous to the previous cases, with the particularity that, for the examples shown in the figure, the V-notched plate has a lower fracture load than that observed in the U-notched plate (the plate with the hole developing the highest critical condition in any case). In Table 2, it can be observed that the V-notches analyzed in this work, with an opening angle of 60°, developed very similar critical loads to the corresponding U-notches, in agreement with the literature (e.g., [19,32]).

**Figure 6:**
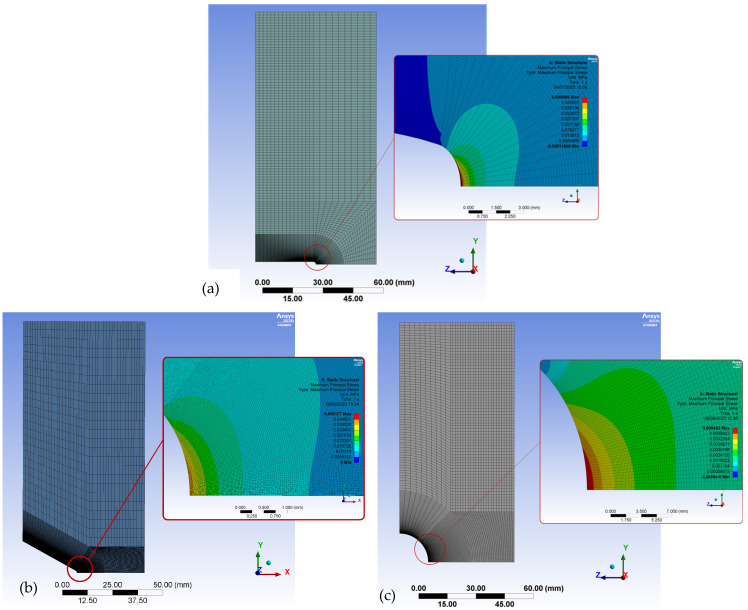
Examples of FE models. Applied load = 1 N in all cases. (**a**) U-notched specimen G302, maximum principal stress = 0.049 MPa; (**b**) V-notched specimen G401, maximum principal stress = 0.050 MPa; (**c**) specimen with central hole G107, maximum principal stress = 0.005 MPa.

**Figure 7:**
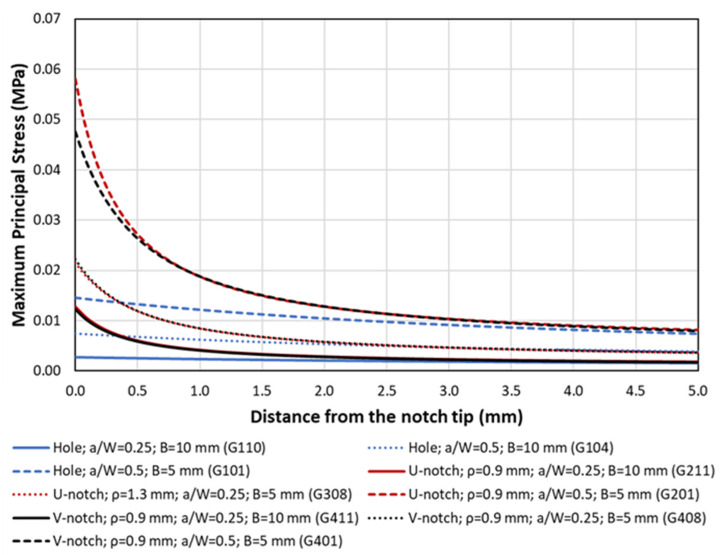
Examples of stress–distance curves obtained at the defect tip. Applied load = 1 N.

**Figure 8:**
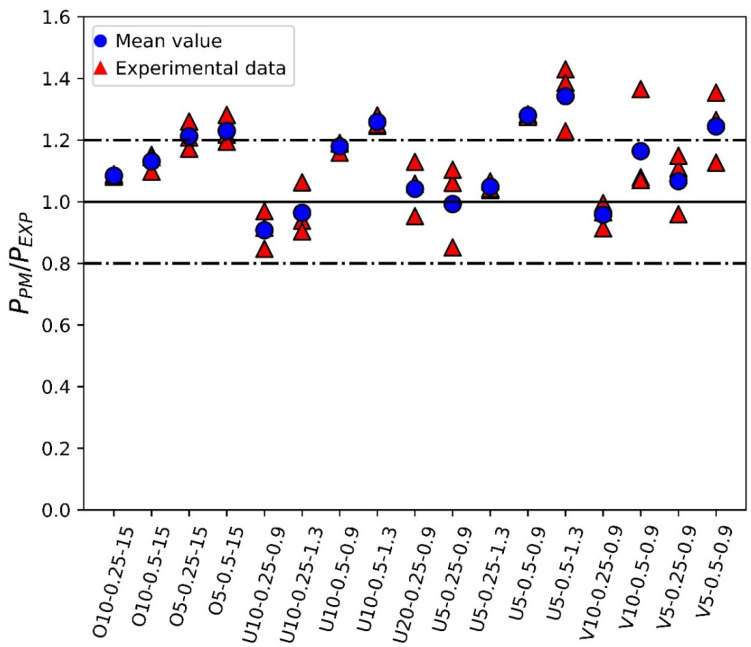
Comparison between fracture load estimations (P_PM_) and experimental fracture loads (P_EXP_), e.g., O10-0.25-15 refers to plate with hole, 10 mm thick, a/W = 0.25, notch radius 15 mm.

Additionally, Figure 6 shows examples of the FE models used in the analysis to derive the corresponding stress field at the notch tip, and Figure 7 represents three of the obtained stress–distance curves for the corresponding unitary load (1N). It can be observed that the stress fields for the U-notches and the V-notches are very similar (justifying their very similar critical loads), whereas the stresses around the holes are clearly lower. Finally, Figure 8 provides a comparison between the experimental critical loads and the obtained estimations.

The results shown in Figure 8 reveal that the proposed approach provides reasonably accurate results for the experimental critical loads. Looking at the comparison between the average experimental values and the PM predictions, most of the results are located within ±20% accuracy, a range generally accepted in fracture analyses bearing in mind the inherent scatter of fracture processes (e.g., [18,19]). There is, however, a certain tendency to overestimate the critical load, with an overall average P_PM_/P_EXP_ ratio of 1.10. This ratio is 1.06 for U-notches, 1.11 for V-notches, and 1.16 for circular holes, which is reasonable if it is considered that the calibration of the PM parameters (L and σ_0_) described in [18] was performed on SENB specimens containing U-notches. Concerning the effect of other geometrical parameters, the best predictions in the three types of defects are achieved for the lower a/W ratio (a/W = 0.25). The highest overestimations are obtained when the a/W = 0.50 ratio is combined with the lower thickness (5 mm). This requires further investigation, considering that the mentioned calibration was performed in 4-mm-thick specimens with a/W = 0.50. Finally, the predictions are equally good for the different nominal notch radii analyzed in this work, which range from 0.9 mm up to 15 mm.

With the aim of explaining the obtained results, an analysis of the fracture surfaces was performed. In the presence of the different types of notches analyzed here, and similarly to the findings of Tse Ng and Susmel [4], cracks initiated at the corresponding notch tip. However, after crack initiation, the subsequent crack propagation took place on material planes that, in general, were not macroscopically perpendicular to the loading direction, resulting from a mixture of different micromechanisms that included the debonding between adjacent/printing layers, the debonding of filaments, and the cracking of the filaments themselves. The detailed micromechanisms observed in the different specimens were as follows.

**Figure 9:**
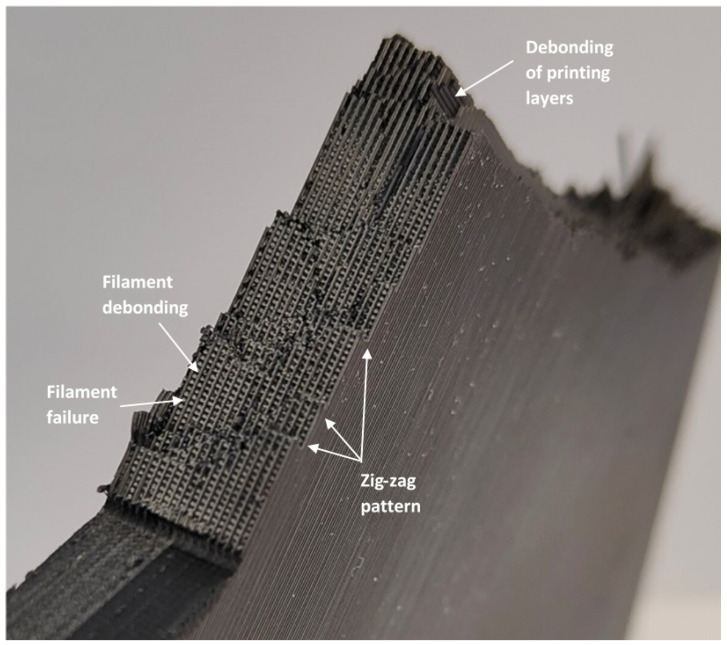
Fracture mechanisms generally observed in U-notches and V-notches, with the image showing U-notched specimen G212.

**Figure 10:**
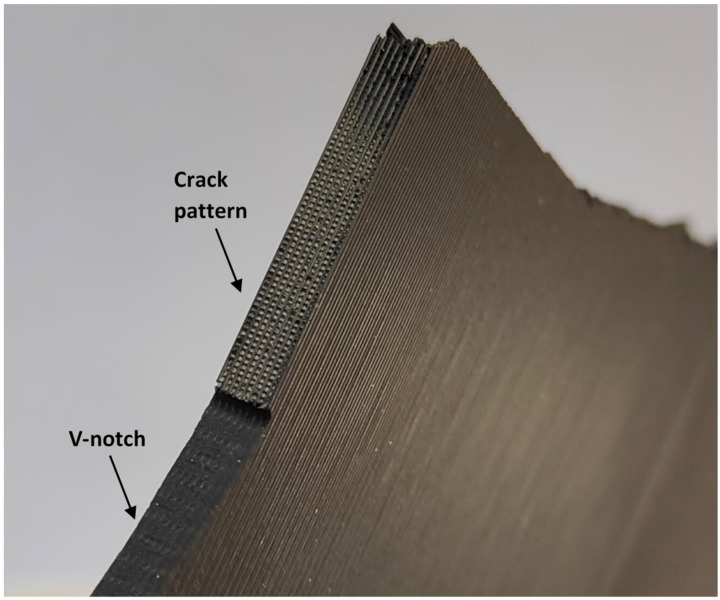
Aspect of the fracture mechanisms observed in U-notched and V-notched 5-mm-thick plates with a/W = 0.25, with the image showing V-notched specimen G408.

**Figure 11:**
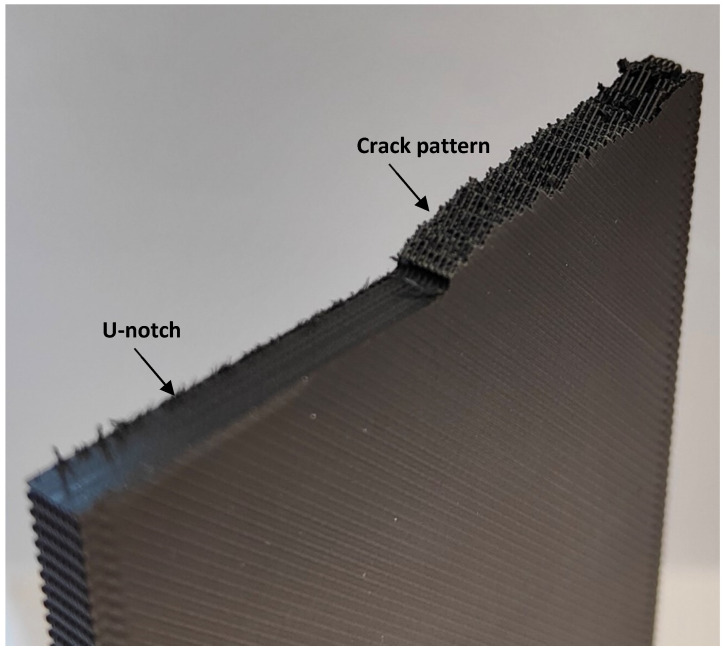
Aspect of the fracture mechanisms observed in U-notches with W = 60 mm and a/W = 0.50. Specimen G201. General crack pattern does not follow the raster orientation.

**Figure 12:**
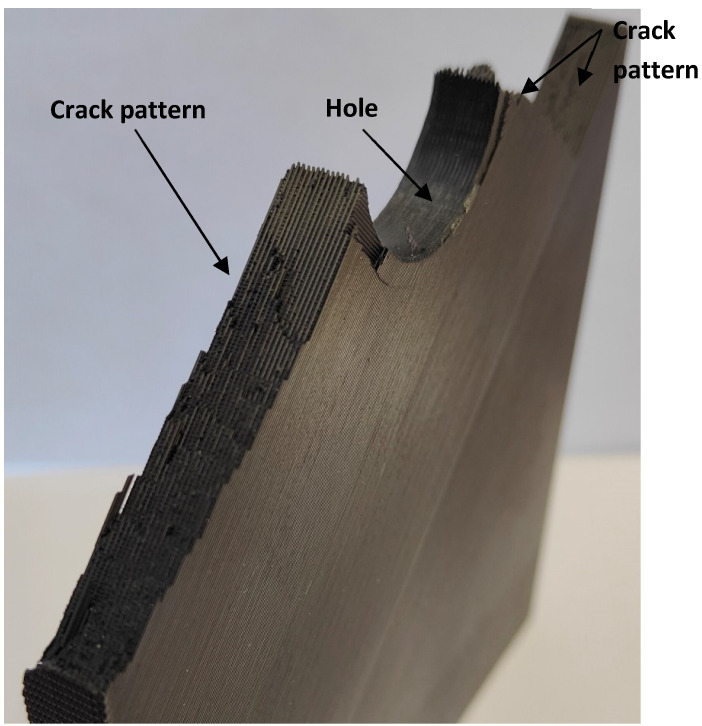
Aspect of the fracture mechanisms observed in specimens with central hole. Specimen G110.

- U-notched and V-notched plates behaved similarly. In both cases, after the peak load, clear macroscopic cracking planes following the raster orientation (+45° or −45°) resulted from a combination of the debonding of the filaments oriented at +45° (or −45°) and the failure of filaments oriented at −45° (or +45°). The initial plane was generally followed by a zig-zag pattern with a dominant macroscopic orientation at −45° (or +45°), as shown in Figure 9. This was observed for the three different thicknesses tested in the experimental program, which represents a certain difference if compared to the fracture behavior of pure PLA specimens, where, as shown in [33], the specimens may present subtle differences for the different thicknesses being considered. The mentioned behavior had two exceptions: firstly, in the case of 5-mm-thick U-notched and V-notched plates with a/W = 0.25, the combination of the filament debonding and the filament failure following the raster orientation did not develop a zig-zag pattern at the initial stages of crack propagation, with the crack extension following a dominant plane (see Figure 10). The accuracy of the critical load predictions for this exception is, however, as good as that obtained in the specimens following the zig-zag mechanisms; secondly, in U-notched specimens with W = 60 mm and a/W = 0.5 (smallest remnant ligament), the macroscopic propagation did not follow the raster orientation and was between this behavior and a flat propagation following the mid-plane of the specimen (Figure 11). The accuracy of the predictions for this second exception was the lowest obtained in the present research, corresponding to the points in Figure 8 associated with the greatest overestimations of the critical loads. This partially explains the worse results obtained for the plates with a/W = 0.5, since some of them developed different fracture mechanisms from those observed in most other geometric conditions. Here, it is important to note that the complex topography of the crack paths makes it very difficult to provide an adequate view of the different fracture processes occurring in the plates, which is the reason for providing these general views of the fractured plates and the fracture surfaces.
- The plates with a central hole, as was the case for the pure PLA material (see [33]), presented similar behavior for the two values of thickness analyzed in this work (5 mm and 10 mm) and the two values of nominal (half) width (30 mm and 60 mm). A fracture is a very complex process resulting from a combination of filament failures, the debonding between filaments, and the debonding between printing layers. The crack patterns do not follow macroscopically the raster orientation. Figure 12 shows an example with a general view of these fracture surfaces.

## 4. Conclusions

The future utilization of 3D (FDM) polymer–matrix composites in structural applications requires the definition of reliable methodologies to estimate the load-carrying capacity of printed parts. With this purpose, this study applied the point method (PM) (and, thus, the Theory of Critical Distances) to estimate critical loads in FDM graphene-reinforced PLA (PLA-Gr) plates containing different types of defects (U-notches, V-notches, and circular holes), and combining a variety of notch radii, thicknesses, and defect length (a) to specimen width (W) ratios. The PM was applied to the stress fields derived from linear–elastic finite element analysis.

The results revealed that the PM provided reasonably accurate predictions of the actual experimental critical loads, with a certain (limited) overestimation of the results (+10% on average) that was more pronounced in central holes (+16%) and much more limited in U-notches (+6%). This was attributed to the fact that the PM material parameters (critical distance, L, and inherent strength, σ_0_) had been calibrated previously on single edge notched bending (SENB) specimens containing U-notches. Moreover, the accuracy of the results was lower in 5-mm-thick specimens with a/W = 0.5, which requires further research in the future, as these are, precisely, the closest conditions to those used in the mentioned calibration.

The fracture behavior was not the same in the different geometries or specimens, although, in all cases, the final fracture resulted from an intricate mixture of micromechanisms, including the debonding between filaments, the debonding between printing layers, and filament failures.

## Figures and Tables

**Table 1:** Mechanical properties of FDM PLA-Gr and the parent FDM PLA material. Raster orientation 45/−45 [18].

Material	E (MPa)	σ_y_ (MPa)	σ_u_ (MPa)	ε_u_ (%)	K_mat_ (MPa·m^1/2^)
PLA	2751 ± 406	35.3 ± 4.6	41.1 ± 5.7	2.6 ± 0.2	4.91 ± 0.25
PLA-Gr	3972 ± 260	47.5 ± 1.4	49.0 ± 2.8	1.5 ± 0.2	7.20 ± 0.31

**Table 2:** Description of the experimental program, including geometrical parameters, experimental critical loads (P_EXP_), and resulting critical load estimations (P_PM_) derived from the PM analysis.

Notch Geometry	Specimen	a (mm)	W (mm)	ρ (mm)	B (mm)	P_EXP_ (kN)	P_PM_ (kN)	P_PM_/P_EXP_
U-notch	G201	30.60	60.51	0.86	4.85	3.87	4.96	1.28
	G202	30.84	60.38	0.91	4.88	3.86	4.99	1.29
	G203	30.73	60.50	0.83	4.85	3.89	4.93	1.26
	G204	30.66	60.46	0.87	10.02	8.52	10.15	1.19
	G205	30.59	60.46	0.88	9.96	8.54	10.15	1.19
	G206	30.83	60.49	0.86	9.98	8.76	10.11	1.15
	G207	31.02	120.36	0.81	4.96	10.55	11.09	1.05
	G208	30.34	120.31	0.83	4.98	13.15	11.19	0.85
	G209	30.58	120.20	0.89	4.97	10.14	11.28	1.11
	G210	31.02	120.36	0.89	10.14	24.43	22.40	0.92
	G211	30.92	120.43	0.84	10.13	26.41	22.28	0.84
	G212	31.06	120.48	0.88	10.00	23.07	22.36	0.97
	G213	31.08	120.43	0.88	20.17	39.90	45.06	1.13
	G214	31.25	120.62	0.89	20.05	42.56	45.08	1.06
	G215	30.83	120.63	0.87	20.14	47.30	45.06	0.95
	G301	30.85	60.48	1.24	4.86	3.69	5.26	1.43
	G302	30.98	60.40	1.24	4.91	4.29	5.26	1.23
	G303	30.91	60.54	1.26	4.77	3.80	5.29	1.39
	G304	30.85	60.47	1.26	9.96	8.63	10.75	1.25
	G305	31.19	60.55	1.27	9.92	8.60	10.77	1.25
	G306	30.95	60.47	1.25	9.93	8.40	10.73	1.28
	G307	30.62	120.32	1.26	4.88	11.51	11.95	1.04
	G308	30.93	120.30	1.27	4.92	11.21	11.97	1.07
	G309	30.92	120.42	1.26	4.94	11.46	12.00	1.05
	G310	31.02	120.25	1.27	9.96	25.37	23.79	0.94
	G311	31.04	120.33	1.26	9.93	22.38	23.77	1.06
	G312	31.08	120.43	1.26	9.93	26.31	23.78	0.90
V-notch	G401	27.03	60.56	1.25	4.76	4.27	5.40	1.26
	G402	26.87	60.54	1.05	4.80	4.09	5.54	1.35
	G403	26.99	60.49	0.89	4.83	4.58	5.16	1.13
	G404	26.95	60.60	0.65	9.92	9.56	10.31	1.08
	G405	26.92	60.55	0.93	9.99	10.04	10.74	1.07
	G406	26.93	60.58	0.87	9.92	8.76	11.96	1.36
	G407	26.95	120.24	1.07	4.89	10.65	11.80	1.11
	G408	26.50	120.26	1.15	4.83	10.30	11.84	1.15
	G409	26.80	120.33	1.01	4.86	12.05	11.56	0.96
	G410	26.96	120.46	0.97	9.94	24.25	23.42	0.97
	G411	26.92	120.29	0.89	9.95	25.32	23.13	0.91
	G412	26.87	120.53	1.05	9.95	24.10	23.99	0.99
Hole	G101	30.39	60.56	15.03	4.85	5.45	6.63	1.22
	G102	30.15	60.36	14.99	4.94	5.18	6.64	1.28
	G103	30.23	60.45	15.04	4.83	5.55	6.61	1.19
	G104	30.18	60.46	14.83	10.04	11.95	13.15	1.10
	G105	30.12	60.58	14.83	9.99	11.38	13.11	1.15
	G106	30.17	60.53	14.87	10.02	11.45	13.13	1.14
	G107	30.22	120.28	14.99	4.86	15.07	17.62	1.17
	G108	30.20	120.35	14.92	4.94	14.62	17.70	1.21
	G109	30.26	120.37	15.04	4.93	14.01	17.69	1.26
	G110	30.14	120.34	14.93	9.98	32.28	34.82	1.08
	G111	30.01	120.23	14.99	9.90	32.23	34.92	1.08
	G112	29.90	120.26	15.01	9.90	32.04	34.93	1.09

## Data Availability

The data presented in this study are available upon request from the corresponding author.

## References

[1] Cantrell J.T., Rohde S., Damiani D., Gurnani R., DiSandro L., Anton J., Young A., Jerez A., Steinbach D., Kroese C. (2017). Experimental Characterization of the Mechanical Properties of 3D-Printed ABS and Polycarbonate Parts. Rapid Prototyp. J..

[2] Bamiduro O., Owolabi G., Haile M.A., Riddick J.C. (2019). The Influence of Load Direction, Microstructure, Raster Orientation on the Quasi-Static Response of Fused Deposition Modeling ABS. Rapid Prototyp. J..

[3] Ahn S., Montero M., Odell D., Roundy S., Wright P.K. (2002). Anisotropic Material Properties of Fused Deposition Modeling ABS. Rapid Prototyp. J..

[4] Ng C.T., Susmel L. (2020). Notch Static Strength of Additively Manufactured Acrylonitrile Butadiene Styrene (ABS). Addit. Manuf..

[5] Ameri B., Taheri-Behrooz F., Aliha M.R.M. (2020). Fracture Loads Prediction of the Modified 3D-Printed ABS Specimens under Mixed-Mode I/II Loading. Eng. Fract. Mech..

[6] Cooper C.A., Ravich D., Lips D., Mayer J., Wagner H.D. (2002). Distribution and Alignment of Carbon Nanotubes and Nanofibrils in a Polymer Matrix. Compos. Sci. Technol..

[7] Hsieh T.H., Kinloch A.J., Masania K., Sohn Lee J., Taylor A.C., Sprenger S. (2010). The Toughness of Epoxy Polymers and Fibre Composites Modified with Rubber Microparticles and Silica Nanoparticles. J. Mater. Sci..

[8] Wan T., Liao S., Wang K., Yan P., Clifford M. (2013). Multi-Scale Hybrid Polyamide 6 Composites Reinforced with Nano-Scale Clay and Micro-Scale Short Glass Fibre. Compos. Part A Appl. Sci. Manuf..

[9] Sreenivasulu B., Ramji B., Nagaral M. (2018). A Review on Graphene Reinforced Polymer Matrix Composites. Mater. Today Proc..

[10] Zhao X., Li Y., Chen W., Li S., Zhao Y., Du S. (2019). Improved Fracture Toughness of Epoxy Resin Reinforced with Polyamide 6/Graphene Oxide Nanocomposites Prepared via in Situ Polymerization. Compos. Sci. Technol..

[11] Zhao X., Chen W., Han X., Zhao Y., Du S. (2020). Enhancement of Interlaminar Fracture Toughness in Textile-Reinforced Epoxy Composites with Polyamide 6/Graphene Oxide Interlaminar Toughening Tackifier. Compos. Sci. Technol..

[12] Cicero S., Parra J.L., Arroyo B., Procopio I. (2020). Graphene Oxide Does Not Seem to Improve the Fracture Properties of Injection Molded PA6. Procedia Struct. Integr..

[13] Murariu M., Dubois P. (2016). PLA Composites: From Production to Properties. Adv. Drug Deliv. Rev..

[14] Rafiee M.A., Rafiee J., Wang Z., Song H., Yu Z.-Z., Koratkar N. (2009). Enhanced Mechanical Properties of Nanocomposites at Low Graphene Content. ACS Nano.

[15] Shen M.-Y., Chang T.-Y., Hsieh T.-H., Li Y.-L., Chiang C.-L., Yang H., Yip M.-C. (2013). Mechanical Properties and Tensile Fatigue of Graphene Nanoplatelets Reinforced Polymer Nanocomposites. J. Nanomater..

[16] Caminero M., Chacón J., García-Plaza E., Núñez P., Reverte J., Becar J. (2019). Additive Manufacturing of PLA-Based Composites Using Fused Filament Fabrication: Effect of Graphene Nanoplatelet Reinforcement on Mechanical Properties, Dimensional Accuracy and Texture. Polymers.

[17] Marconi S., Alaimo G., Mauri V., Torre M., Auricchio F. (2017). Impact of Graphene Reinforcement on Mechanical Properties of PLA 3D Printed Materials. Proceedings of the 2017 IEEE MTT-S International Microwave Workshop Series on Advanced Materials and Processes for RF and THz Applications (IMWS-AMP).

[18] Cicero S., Martínez-Mata V., Castanon-Jano L., Alonso-Estebanez A., Arroyo B. (2021). Analysis of Notch Effect in the Fracture Behaviour of Additively Manufactured PLA and Graphene Reinforced PLA. Theor. Appl. Fract. Mech..

[19] Taylor D. (2007). The Theory of Critical Distances: A New Perspective in Fracture Mechanics.

[20] Taylor D. (2004). Predicting the Fracture Strength of Ceramic Materials Using the Theory of Critical Distances. Eng. Fract. Mech..

[21] Cicero S., Madrazo V., Carrascal I.A. (2012). Analysis of Notch Effect in PMMA Using the Theory of Critical Distances. Eng. Fract. Mech..

[22] Negru R., Marsavina L., Voiconi T., Linul E., Filipescu H., Belgiu G. (2015). Application of TCD for Brittle Fracture of Notched PUR Materials. Theor. Appl. Fract. Mech..

[23] Cicero S., Madrazo V., Carrascal I.A. (2012). On the Point Method Load-Bearing Capacity Predictions in Al7075-T651 Structural Components Containing Stress Risers. Eng. Fail. Anal..

[24] Marsavina L., Sapora A., Susmel L., Taylor D. (2023). The Application of the Theory of Critical Distances to Nonhomogeneous Materials. Fatigue Fract. Eng. Mater. Struct..

[25] Ahmed A.A., Susmel L. (2017). On the Use of Length Scale Parameters to Assess the Static Strength of Notched 3D-Printed PLA. Frat. Ed Integrità Strutt..

[26] Shahbaz S., Ayatollahi M.R., Petru M., Torabi A.R. (2022). U-Notch Fracture in Additively Manufactured ABS Specimens under Symmetric Three-Point Bending. Theor. Appl. Fract. Mech..

[27] Schmeier G.E.C., Tröger C., Kwon Y.W., Sachau D. (2023). Predicting Failure of Additively Manufactured Specimens with Holes. Materials.

[28] (2014). Standard Test Method for Tensile Properties of Plastics.

[29] (2010). Standard Test Method for Determining J-R Curves of Plastic Materials.

[30] Neuber H. (1958). Therory of Notch Stresses: Principles for Exact Calculation of Strength with Reference to Structural Form and Material.

[31] Peterson R.E., Sines G., Waisman J.L. (1959). Notch Sensitivity. Metal fatigue.

[32] Lazzarin P., Zambardi R. (2001). A finite-volume-energy based approach to predict the static and fatigue behavior of components with sharp V-shaped notches. Int. J. Fract..

[33] Cicero S., Arrieta S., Sánchez M., Castanon-Jano L. (2023). Analysis of Additively Manufactured Notched PLA Plates Using Failure Assessment Diagrams. Theor. Appl. Fract. Mech..

